# A 3-Marker Index Improves the Identification of Iron Disorders in CKD Anaemia

**DOI:** 10.1371/journal.pone.0084144

**Published:** 2014-02-19

**Authors:** Lucile Mercadal, Marie Metzger, Jean Philippe Haymann, Eric Thervet, Jean-Jacques Boffa, Martin Flamant, François Vrtovsnik, Cédric Gauci, Marc Froissart, Bénédicte Stengel, on behalf of the NephroTest Study Group

**Affiliations:** 1 Institut National de la Santé et de la Recherche Médicale U1018, CESP Centre for research in Epidemiology and Population Health, Epidemiology of Diabetes, Obesity, and Kidney Diseases Team, Villejuif, France; 2 Department of Nephrology, Hôpital Pitié-Salpêtrière, Assistance Publique-Hôpitaux de Paris, Paris, France; 3 Univ Paris Sud 11, UMRS 1018, Villejuif, France; 4 Department of Physiology and Nephrology, Hôpital Tenon, Assistance Publique-Hôpitaux de Paris, Université Pierre et Marie Curie, Paris, France; 5 Institut National de la Santé et de la Recherche Médicale U702, Paris, France; 6 Department of Nephrology, Hôpital Européen G Pompidou, Assistance Publique-hôpitaux de Paris, Université Paris Descartes, Paris, France; 7 Institut National de la Santé et de la Recherche Médicale UMR S775, Paris, France; 8 Department of Nephrology, Hôpital Tenon, Assistance Publique-hôpitaux de Paris, Université Pierre et Marie Curie, Paris, France; 9 Department of Physiology, Hôpital Bichat, Assistance Publique-hôpitaux de Paris, Paris, France; 10 Institut National de la Santé et de la Recherche Médicale U699, Université Paris Diderot, Paris, France; 11 Department of Nephrology, Hôpital Bichat, Assistance Publique-hôpitaux de Paris, Paris, France; 12 Department of Physiology, Hôpital Européen G Pompidou, Assistance Publique-hôpitaux de Paris, Université Paris Descartes, Paris, France; National Institute of Medical Research, United Kingdom

## Abstract

**Background:**

Iron disorders are common and complex in chronic kidney disease (CKD). We sought to determine whether a 3-marker index would improve the classification of iron disorders in CKD anaemia.

**Methods:**

We studied the association between Hb level and iron indexes combining 2 or 3 of the following markers: serum ferritin (<40 ng/mL), transferrin saturation (TSAT<20%) and total iron binding capacity (TIBC<50 µmol/L) in 1011 outpatients with non-dialysis CKD participating in the Nephrotest study. All had glomerular filtration rates measured (mGFR) by ^51^Cr-EDTA renal clearance; 199 also had hepcidin measures.

**Results:**

The TSAT-TIBC-ferritin index explained Hb variation better than indexes combining TSAT-TIBC or ferritin-TSAT. It showed hypotransferrinaemia and non-inflammatory functional iron deficiency (ID) to be more common than either absolute or inflammatory ID: 20%, 19%, 6%, and 2%, respectively. Hb was lower in all abnormal, compared with normal, iron profiles, and decreased more when mGFR was below 30 mL/min/1.73 m^2^ (interaction p<0.0001). In patients with mGFR<30 mL/min/1.73 m^2^, the Hb decreases associated with hypotransferrinaemia, non-inflammatory functional ID, and absolute ID were 0.83±0.16 g/dL, 0.51±0.18 and 0.89±0.29, respectively. Compared with normal iron profiles, hepcidin was severely depressed in absolute ID but higher in hypotransferrinaemia.

**Conclusions:**

The combined TSAT-TIBC-ferritin index identifies hypotransferrinaemia and non-inflammatory functional ID as the major mechanisms of iron disorders in CKD anaemia. Both disorders were associated with a greater decrease in Hb when mGFR was <30 mL/min/1.73 m^2^. Taking these iron profiles into account may be useful in stratifying patients in clinical trials of CKD anaemia and might improve the management of iron therapy.

## Introduction

Anaemia is an early complication of chronic kidney disease (CKD) [1], associated with symptoms, potential need for blood transfusion and increased morbidity and mortality [2]. Relative erythropoietin (EPO) deficiency occurs rapidly with kidney function decline and is the main determinant of anaemia in advanced CKD (GFR<30 mL/min/1.73 m^2^) [3]. The efficacy of erythropoietin-stimulating agents (ESA), however, depends highly on iron bioavailability. Iron metabolism disorders are common and complex in CKD, but few studies have investigated their relations with anaemia in early-stage CKD [4], [5], [6]. Two of these studies examined the relations between Hb and both transferrin saturation (TSAT) and ferritin. One showed bone marrow iron decreased as TSAT dropped, at thresholds of 25, 20 and 15% and as ferritin fell below 100 and 75 µg/l [4]. In the other study, anaemia was only related with TSAT [5]. Two other studies have also showed that iron disorders modify ESA response [6], [7].

Clinical nephrology guidelines recommend use of serum ferritin and TSAT measure to guide iron therapy [8]. In haematology, however, the panel of blood tests to assess iron status also includes transferrin and total iron-binding capacity (TIBC), the latter directly derived from transferrin (TIBC = 25×transferrin (g/L)). These results are usually combined to define different iron status profiles. The combined TIBC-TSAT index is used to discriminate iron deficiency (ID) from inflammatory syndrome related anaemia [9], while combining serum ferritin and TSAT [10] differentiates absolute *vs* functional ID (table 1). Each of these parameters represents an iron compartment. Ferritin measures iron stores. Transferrin is the main blood iron transporter and is required for the internalization of iron into the red blood cell precursors. TSAT, calculated as free iron over TIBC, assesses the blood iron content. Low iron stores together with low blood iron content define absolute iron deficiency, while high iron stores together with low blood iron content define functional iron deficiency. In this condition, the iron stores cannot be used. Finally, a transferrin deficiency with normal iron content has never been studied separately, but may also affect both iron use and Hb level. We therefore sought to determine whether combining these three iron tests would improve the identification of iron metabolism disorders as compared with the TSAT-ferritin index routinely used in the management of CKD anaemia.

**Table 1:** Iron status index combining ferritin, transferrin saturation (TSAT) and total iron-binding capacity (TIBC).

	Threshold 20.50.40			Threshold 15.45.20			Threshold 25.55.100		
Iron Indices	TSAT %	TIBC µmol/L	Ferritin ng/ml	TSAT %	TIBC µmol/L	Ferritin ng/ml	TSAT %	TIBC µmol/L	Ferritin ng/ml
**TSAT-Ferritin index ^10^**									
Normal	≥20	-	-	≥15	-	-	≥25	-	-
Absolute iron deficiency	<20	-	<40	<15	-	<20	<25	-	<100
Functional iron deficiency	<20	-	≥40	<15	-	≥20	<25	-	≥100
**TSAT-TIBC index**									
Normal	≥20	≥50	-	≥15	≥45	-	≥25	≥55	-
Iron deficiency	<20	≥50	-	<15	≥45	-	<25	≥55	-
Inflammatory iron deficiency	<20	<50	-	<15	<45	-	<25	<55	-
Hypotransferrinaemia	≥20	<50	-	≥15	<45	-	≥25	<55	-
**TSAT-TIBC-ferritin index**									
Normal	≥20	≥50	-	≥15	≥45	-	≥25	≥55	-
Absolute iron deficiency	<20	≥50	<40	<15	≥45	<20	<25	≥55	<100
Noninflammatory functional iron deficiency	<20	≥50	≥40	<15	≥45	≥20	<25	≥55	≥100
Inflammatory functional iron deficiency	<20	<50	≥40	<15	<45	≥20	<25	<55	≥100
Hypotransferrinaemia	≥20	<50	-	≥15	<45	-	≥25	<55	-

*Each combination of iron marker and their threshold values is tested in this study.*

Total iron-binding capacity (TIBC) in µmol/L was calculated as 25×transferrin (g/L).

Transferrin saturation (TSAT) was calculated as serum iron/TIBC×100%.

We therefore compared the relations of various combinations of serum ferritin, TSAT and TIBC with haemoglobin level in 1011 patients with non-dialysis CKD to identify the combined index with the strongest impact on haemoglobin, independent of other anaemia risk factors. We also studied trends in these associations with kidney function decline.

## Methods

### Population

NephroTest is a prospective hospital-based cohort study, enrolling adult outpatients with all diagnoses of CKD stages 1 to 5, who were not pregnant, not on dialysis or living with a kidney transplant, and who had been referred to any of three physiology departments for extensive clinical and laboratory work-ups [1]. All patients signed an informed consent at inclusion. Of 1,294 NephroTest patients included between January 2000 and December 2009, we excluded 78 who were treated with ESA or intravenous iron, 125 without treatment information, and 80 with missing Hb or iron test values. This analysis thus covered 1011 patients. The NephroTest study design was approved by the relevant ethics committee (DGRI CCTIRS MG/CP09.503).

### Laboratory measures

Glomerular filtration rates were measured (mGFR) by renal clearance of ^51^Cr-EDTA in all patients. We also measured Hb, mean corpuscular volume, serum albumin, serum folate, C-reactive protein, and urinary protein-to-creatinine ratio (UPCR). EPO was measured in a subgroup of 251 patients and hepcidin in 188 patients; 152 had both measurements. Endogenous EPO levels were determined in serum (100 µL) with the Quantitine IVD Epo double-antibody sandwich ELISA method from R&D Systems (Minneapolis, MN). Hepcidin was measured by an electrochemiluminescent test developed at Amgen, Thousand Oaks, CA [10].

### Assessment of iron status

Serum iron (DxC800 Beckman-Coulter, ferrozine, emitted light 560 nm), ferritin (BN-Siemens, N-latex ferritin immunonephelometry), and transferrin (BN-Siemens, N Antiserum antitransferrin immunonephelometry) were measured in all patients. TIBC (µmol/L) was calculated as 25×transferrin (g/L). TSAT (%) was calculated as serum iron×100/TIBC.

We studied three different iron indexes: the combined ferritin-TSAT and TIBC-TSAT indexes described above [9], [10], and one combining ferritin, TSAT and TIBC. The latter discriminated five profiles: normal, absolute ID, non-inflammatory functional ID, inflammatory functional ID, and hypotransferrinaemia (Table 1). For each index, we studied three different cut-off points. TSAT<20% and TIBC<50 µmol/L were chosen first, as the lower limit of these normal values, and ferritin<40 ng/mL, because it is the level usually recommended for diagnosing absolute ID [10]. We then used more specific or more sensitive definitions: ferritin<20 ng/ml, TSAT<15% and TIBC<45 µmol/L; and ferritin<100 ng/ml, TSAT<25%, and TIBC<55 µmol/L.

### Statistical analyses

We first studied factors associated with Hb levels, treated as a continuous or a categorical variable, by gender. Anaemia was defined according to either KDOQI (Hb<11 g/dL) or WHO gender-specific criteria (Hb<12/13 g/dL in women/men). Crude associations were analyzed with the Kruskal-Wallis and Cochran-Armitage tests, as appropriate. Secondly, we plotted levels of mean Hb and of each iron marker according to mGFR level (≥60, 45–59, 30–44, 15–29, and <15 mL/min/1.73 m^2^), by gender. Linear regression models were used to test interactions with gender in the relations between mGFR and these markers. Because ferritin is not normally distributed, this variable was log-transformed in this analysis (Figure S1).

We then ran multivariate regression analyses to study the effect of the different iron markers alone or in combinations of 2 or 3 markers on Hb concentration after adjusting for well-established anaemia risk factors and potential confounders [11]. Variables that were not associated with Hb (p>0.2) were not included in final model. Moreover, we verified that correlation coefficients between independent variables were less than 0.5 to avoid any problem of colinearity in our models.

The best iron index was defined as the one producing the Hb model with the best fit after adjustment for confounders. We used the Bayesian Information Criterion (BIC or Schwartz criteria) and the Akaike information Criterion (AIC) to compare non-nested models. The model with the lowest BIC or AIC is considered the best.

We additionally tested interactions with both gender and mGFR, treated as a continuous or a categorical variable < or ≥30 mL/min/1.73 m^2^ in the relations between Hb and the iron indexes. Similarly, to validate the relevance of the various levels chosen (ferritin 40 ng/ml, TSAT 20%, and TIBC 50 µmol/L), we compared models including iron indexes with more specific or more sensitive definitions, as described above.

Finally, we studied patient characteristics and measurements according to the best combined iron index. We used ANOVA to compare quantitative variables and logistic regression for qualitative ones and defined statistical significance as P<0.05. We performed statistical analyses with SAS 9.2 (SAS Institute, Cary, NC).

## Results

### Patient characteristics

Table 2 summarizes the characteristics of the NephroTest cohort patients by gender. More than half had WHO-defined anaemia. Vascular nephropathy, primary glomerulonephritis, and diabetic nephropathy (either biopsy-proven or defined by a history of albuminuria >300 mg/g, or creatininuria or retinopathy or/and neuropathy) accounted for two thirds of the cases.

**Table 2:** Patient characteristics by gender.

	Overall	Men	Women
**No of patients**	1011	718	293
**Age (Years), mean ± sd**	60.2±14.7	60.7±14.6	58.9±14.9
**Sub-Saharan African origin, %**	8.1	10.2	4.3
**Diabetes mellitus, %**	28.0	29.9	23.2
with diabetic glomerulopathy	15.2	16.7	11.6
with other nephropathy types	12.8	13.2	11.6
**Body Mass Index (kg/m^2^), mean ± sd**	26.5±4.9	26.7±4.5	25.9±5.9
**Systolic BP mmHg, median [IQR]**	136 [124–150]	138 [125–152]	132 [118–148]
**Diastolic BP mmHg, median [IQR]**	75 [68–83]	76 [69–84]	72 [65–79]
**mGFR mL/min/1.73 m^2^, median [IQR]**	35.9 [25.6–48.9]	37.4 [27.1–51.0]	34.6 [23.9–49.5]
**CKD stages, %**			
1–2 (mGFR≥60 ml/min/1.73 m^2^)	15.3	15.5	15.0
3a (45–60)	19.3	20.6	16.0
3b (30–45)	31.0	32.0	28.3
4 (15–30)	28.2	25.9	33.8
5 (<15)	6.2	6.0	6.8
**eGFR CKDEPI mL/min/1.73 m^2^, median [IQR]**	36.9 [25.9–50.8]	37.2 [26.3–51.5]	35.9 [24.9–49.7]
**UPCR mg/mmol, median [IQR]**	32.3 [13.5–113.5]	33.7 [12.8–117.8]	31.6 [15.0–102.0]
**Total cholesterol (mmol/L), mean ± sd**	5.0±1.2	4.9±1.2	5.3±1.2
**Serum albumin (g/L), mean ± sd**	39.3±5.0	39.8±5.0	38.2±4.6
**C-reactive protein>8 mg/L, %**	12.0	12.0	11.9
**Hb (g/dL), mean ± sd**	12.6±1.6	12.9±1.6	11.8±1.4
**WHO anaemia, %**	53.1	51.9	56.0
**Ferritin ng/mL, median [IQR]**	128.8 [68.2–219.0]	155.1 [81.2–238.8]	91.2 [41.9–155.8]
**TSAT, %**	26.1±10.0	26.7±10.0	24.4±9.7
**TIBC µmol/L, mean ± sd**	56.7±10.6	56.3±10.2	57.6±11.5
**Oral iron therapy, %**	9.0	6.4	15.4
**RASi, %**	79.1	82.2	71.7

*Abbreviations*: BP, blood pressure; mGFR, measured glomerular filtration rate; eGFR CKDEPI, estimated glomerular filtration rate using the Chronic Kidney Disease Epidemiology Collaboration equation; UPCR, urinary protein-to-creatinine ratio; Hb, haemoglobin; WHO-anaemia, defined according to World Health Organization as Hb level <13 g/dL for men (<12 g/dL for women); TSAT, transferrin saturation; TIBC, transferrin iron-binding capacity; RASi, renin-angiotensin system inhibitors. TIBC (µmol/L) was calculated as 25×transferrin (g/L). TSAT was calculated as serum iron/TIBC×100%.

### Factors associated with haemoglobin level and anaemia, by gender

Hb significantly decreased with age in men but not in women (Table 3). For both genders, Sub-Saharan African origin, diabetic nephropathy, lower mGFR, higher proteinuria and higher CRP levels were associated with lower Hb levels and higher prevalence of anemia. In men, low serum albumin was also associated with higher anaemia prevalence. Of note, there was an inverse association between serum folate and Hb. Hb levels did not differ between patients treated with either single or double RAS blockade or folate and those not so treated, but mean Hb was slightly lower in patients receiving oral iron therapy or diuretics (data not shown).

**Table 3:** Factors associated with haemoglobin level and anaemia by gender.

		Men							Women						
		N	mean ± sd	p-value	WHO anaemia%	p-value	KDOQI anaemia%	p-value	N	mean ± sd	p-value	WHO anaemia%	p-value	KDOQI anaemia%	p-value
**Age classes**	<55 yr	230	13.2±1.8	0.001	43	0.005	12.2	0.8	111	11.7±1.5	0.8	57.7	0.95	27	0.7
	55–70 yr	257	12.8±1.5		56		10.1		95	11.8±1.3		51.6		30.5	
	≥70 yr	231	12.7±1.5		56.3		11.3		87	11.7±1.3		58.6		24.1	
**African origin**	No	616	12.9±1.6	0.01	51.1	0.2	9.4	<0.0001	265	11.8±1.4	0.1	54	0.04	26	0.2
	Yes	70	12.7±1.7		54.3		15.7		12	11.2±1.1		75		41.7	
	Unknown	32	12.1±1.8		62.5		34.4		16	11.4±1.2		75		37.5	
**Diabetic nephropathy**	No without DM	503	13.0±1.6	<0.0001	48.3	<0.0001	10.1	0.02	225	11.8±1.4	0.007	54.7	0.2	25.8	0.05
	No with DM	120	13.0±1.4		50		7.5		34	11.9±1.2		50		17.6	
	Yes	95	12.1±1.5		73.7		21.1		34	11.1±1.3		70.6		47.1	
**BMI**	<25	263	12.7±1.6	0.02	54.8	0.07	11	0.8	153	11.7±1.4	0.9	58.2	0.3	29.4	0.5
	25–30	312	12.9±1.6		52.9		11.9		75	11.8±1.3		56		24	
	≥30	143	13.2±1.6		44.8		9.8		65	11.8±1.4		50.8		26.2	
**mGFR (mL/min/1.73 m^2^)**	≥60	111	13.9±1.5	<0.0001	25.2	<0.0001	1.8	<0.0001	44	12.2±1.3	<0.0001	40.9	<0.0001	11.4	<0.0001
	45–60	148	13.4±1.4		34.5		3.4		47	12.3±1.0		42.6		6.4	
	30–45	230	12.9±1.5		52.2		8.7		83	11.9±1.4		50.6		26.5	
	15–30	186	12.1±1.3		72.6		18.8		99	11.3±1.3		69.7		43.4	
	<15	43	11.2±1.4		90.7		41.9		20	11.2±1.6		75		35	
**UPCR (mg/mmol)**	<30	319	13.2±1.5	<0.0001	42.3	0.01	6.3	0.04	133	12.1±1.2	0.001	45.1	0.01	17.3	0.01
	30–300	288	12.6±1.6		59.4		15.6		116	11.5±1.5		63.8		36.2	
	> = 300	60	12.2±1.4		71.7		15		23	11.6±1.3		69.6		30.4	
	missing	51	12.9±1.9		47.1		11.8		21	11.4±1.2		66.7		38.1	
**Albuminaemia(g/L)**	<40	355	12.6±1.6	<0.0001	61.1	<0.0001	14.4	0.006	191	11.6±1.4	0.02	59.7	0.1	30.9	0.08
	≥40	357	13.2±1.6		42.9		7.8		99	12.0±1.2		49.5		21.2	
**C-Reactive Protein (mg/L)**	<8	609	13.0±1.6	0.0011	49.8	0.03	10	0.06	248	11.8±1.4	0.009	52.4	0.005	25	0.01
	≥8	86	12.3±1.5		67.4		18.6		35	11.4±1.1		74.3		34.3	
	missing	23	12.5±1.4		52.2		13		10	10.8±1.4		80		60	
**Folates (ng/mL)**	<5.4	251	13.2±1.6	<0.0001	46.2	0.0002	8	0.006	74	11.9±1.6	0.003	47.3	0.0006	29.7	0.5
	5.4–8.3	224	13.1±1.5		46.4		8.1		96	12.0±1.3		45.8		18.9	
	≥8.3	216	12.4±1.6		63		18.1		115	11.4±1.2		68.4		33	
	missing	27	12.7±1.3		63		7.4		9	11.4±1.0		77.8		22.2	
**Current smoking**	No	627	12.8±1.6	0.1	52.6	0.3	11.2	0.96	256	11.7±1.4	0.2	57.8	0.1	27.7	0.7
	Yes	91	13.1±1.7		47.3		11		37	12.0±1.5		43.2		24.3	
**Oral iron therapy**	No	672	13.0±1.5	<0.0001	49.4	<0.0001	8.8	<0.0001	248	11.9±1.3	<0.0001	51.6	0.0004	22.6	<0.0001
	Yes	46	11.2±1.6		89.1		45.7		45	11.0±1.3		80		53.3	

Anaemia, defined according to World Health Organization as hemoglobin level <13 g/dL for men and <12 g/dL for women; defined according Kidney Disease Outcomes Quality Initiative as hemoglobin level <11 g/dL. **Abbreviations**: DM, diabetes mellitus; mGFR, measured glomerular filtration rate; UPCR, urinary protein-creatinine ratio;

### Levels of haemoglobin and iron markers according to mGFR level, by gender

**Figure 1:**
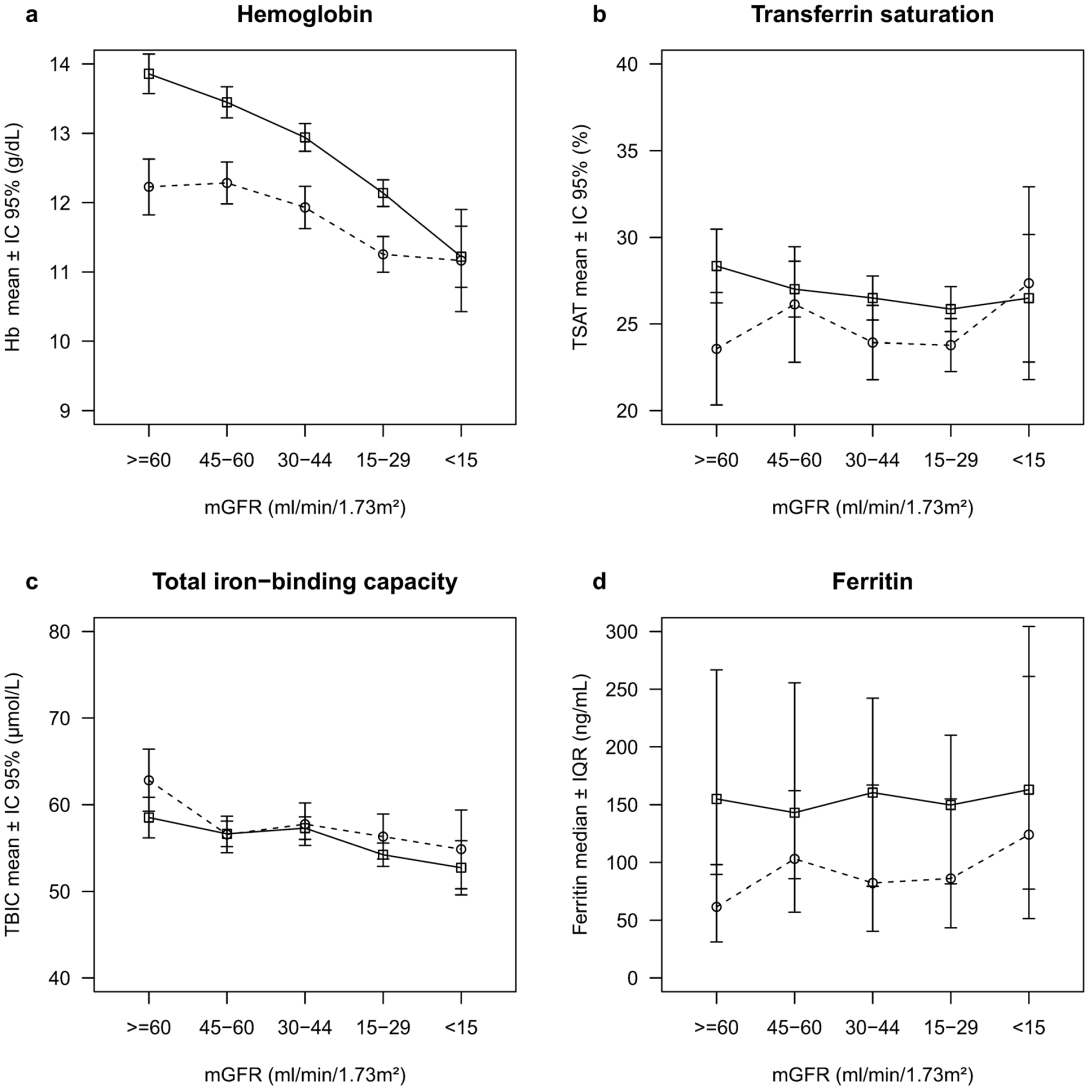
Hemoglobin, transferrin saturation (TSAT), ferritin and transferrin iron binding capacity (TIBC) according to mGFR level, by gender. Men are in solid line and women in dotted line.

Hb levels decreased faster with mGFR decline in men (interaction p = 0.001) than women, and reached the levels in women at mGFR<15 mL/min/1.73 m^2^ (Figure 1). The relation between ferritin and mGFR varied by gender (interaction p = 0.01), increasing with decreasing mGFR only in women (p = 0.008). The TSAT level was lower in women than men (p = 0.001), but did not change with mGFR (p = 0.2). In contrast, TIBC was slightly higher in women (p = 0.02) and decreased similarly and significantly with mGFR decline in both genders (p<0.0001).

### Relations between iron metabolism indexes and Hb level

There was no interaction with gender in the relations between Hb and any of the iron indexes. In contrast, there were significant interactions with mGFR in the relations between Hb and each iron test as well as the combined indexes (all p<0.001). All multivariate analyses were therefore run by mGFR level (Table 4), but irrespective of gender. All abnormal iron status profiles were associated with a greater Hb decrease than the normal status when mGFR was below 30 mL/min/1.73 m^2^. The Hb decline for absolute ID, non-inflammatory functional ID, and hypotransferrinaemia, defined by the 20-50-40 TSAT-TIBC-ferritin index, was 0.89±0.29, 0.51±0.18 and 0.83±0.16 g/dL under 30 mL/min/1.73 m^2^, respectively (Table 4). Of all the iron indexes we tested, this index produced the Hb model with the lowest AIC value (Table 4). Using a more specific definition with thresholds of 15-45-20, respectively, produced a much higher BIC, as did using a more sensitive definition with thresholds of 25-55-100 (BIC = 3530, data not shown). However, AIC values were close between the *best* model and the model including the TSAT-TIBC index with the 20–50 thresholds. The BIC values for the relation of Hb levels to ferritin, TSAT and TIBC, considered separately, were substantially higher (all above 3530, data not shown) than that for the combined TSAT-TIBC-ferritin index. Therefore, the combined TSAT-TIBC-ferritin index, with thresholds of 20%, 50 µmol/L and 40 ng/mL, was the one with the strongest association with Hb decreases.

**Table 4:** Multivariate analyses of haemoglobin changes (in g/dL) according to different definitions of iron status profile, stratified by mGFR classes.

Iron indexes^1^	N	BIC/AIC^2^ (n = 1011)	mGFR in mL/min/1.73 m^2^			
			≥30 (n = 663)		<30 (n = 348)	
			β±sd^3^	p-value	β±sd^3^	p-value
**TSAT-TIBC-ferritin index 20.50.40**		3516/3363		<0.0001		<0.0001
Normal	538		Ref		Ref	
Absolute iron deficiency	62		−0.84±0.22	<0.0001	−0.89±0.29	0.002
Non inflammatory functional ID	188		−0.43±0.14	0.001	−0.51±0.18	0.004
Inflammatory ID	21		−0.97±0.37	0.009	−0.46±0.38	0.2
Hypotransferrinaemia	202		−0.44±0.14	0.002	−0.83±0.16	<0.0001
**TSAT-TIBC-ferritin index 15.45.20**		3524/3372		0.001		<0.0001
Normal	825		Ref		Ref	
Absolute iron deficiency	19		−1.09±0.36	0.003	−2.26±0.51	<0.0001
Non inflammatory functional ID	70		−0.30±0.20	0.1	−0.70±0.26	0.007
Inflammatory ID	2		−0.24±0.90	0.8	−1.94±1.20	0.1
Hypotransferrinaemia	95		−0.61±0.20	0.002	−0.85±0.19	<0.0001
**TSAT-TIBC iron index 20.50**		3507/3364		<0.0001		<0.0001
Normal	538		Ref		Ref	
Iron deficiency	250		−0.53±0.12	<0.0001	−0.60±0.16	0.0002
Inflammatory ID	21		−0.96±0.37	0.01	−0.46±0.38	0.2
Hypotransferrinaemia	202		−0.44±0.14	0.002	−0.84±0.16	<0.0001
**TSAT-TIBC iron index 15.45**		3520/3377		0.0006		<0.0001
Normal	825		Ref		Ref	
Iron deficiency	89		−0.44±0.18	0.02	−1.01±0.24	<0.0001
Inflammatory ID	2		−2.66±1.27	0.04	−1.92±1.22	0.1
Hypotransferrinaemia	95		−0.61±0.20	0.002	−0.84±0.20	<0.0001

*Abbreviations:* BIC Bayesian Information Criterion; AIC, Akaike information Criterion. TSAT, transferrin saturation; TIBC, transferrin iron-binding capacity; ID, iron deficiency; Ref, reference class.

1 See BOX 1 for definitions of iron indexes.

2 BIC and AIC were given for the multivariate model taking into account the interaction term between mGFR and iron tests. Best values for BIC and AIC are underlined.

3 Regression coefficients for the different iron indexes in the linear regression models of Hb levels, stratified by mGFR classes. Models are adjusted for age, gender, ethnicity, smoking, diabetic nephropathy, renin angiotensin system inhibitors and oral iron use, C-reactive protein, serum folic acid, serum albumin, and centre.

### Prevalence of iron metabolism disorders by gender and associated factors

**Figure 2:**
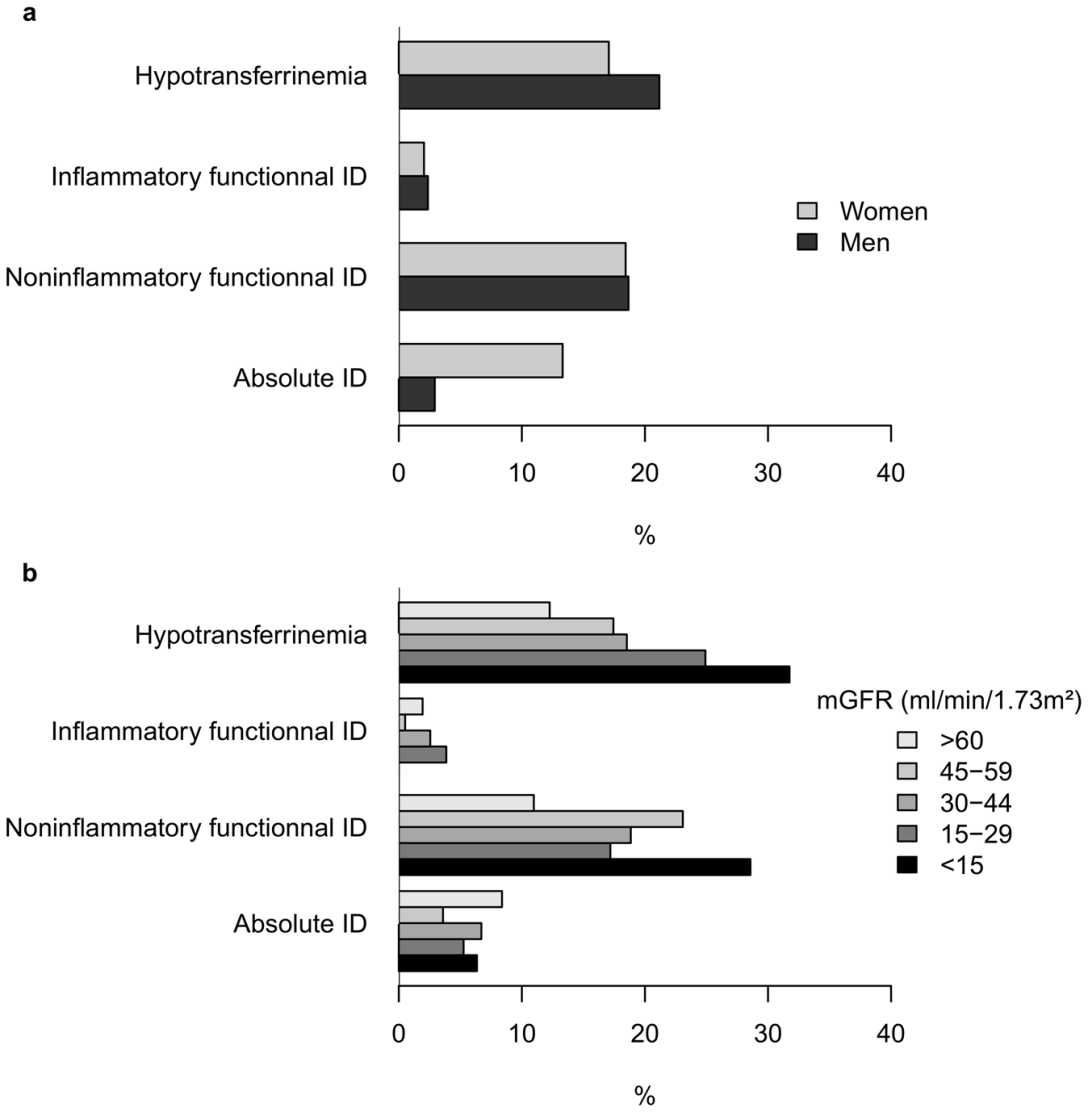
Iron profiles distribution according to gender (2a) and mGFR (2b).

The overall prevalences of each iron metabolism disorder as defined by the TSAT-TIBC-ferritin combined index were as follows: 6% for absolute ID, 19% for non-inflammatory functional ID, 2% for inflammatory functional ID, and 20% for isolated hypotransferrinaemia (Table 5). These prevalences were similar in men and women, except for absolute ID, which was four times more common in women (13.3%) than men (2.9%) (Figure 2a). Prevalences of non-inflammatory functional ID and hypotransferrinaemia steadily increased with decreasing mGFR, while that of absolute ID did not (Figure 2b). Compared with patients with no iron disorder, those with non-inflammatory functional ID had higher BMI and CRP values and lower serum albumin levels (Table 6). Patients with hypotransferrinaemia were younger, had lower BMI and serum albumin levels, and higher proteinuria and CRP levels.

**Table 5:** Distribution of patients according to the TSAT-ferritin index [10] (rows), the TSAT-TIBC index (columns) and the combined TSAT-TIBC-ferritin index (cells).

		TSAT-TIBC index				
		Normal	Iron deficiency	Inflammatory syndrome	Hypotransferrinaemia	Total
**TSAT- ferritin index**	Normal	**538 (53.2)**	0	0	**202 (19.9)**	740
	Absolute ID	0	**60 (5.9)**	2 (0.2)	0	62
	Functional ID	0	**188 (18.6)**	**21 (2.1)**	0	209
	Total	538	248	23	202	1011

Data are expressed as n (%).

Cells include numbers (%) for the five iron profiles defined by the combined ferritin-TSAT-TIBC index : normal, absolute ID, noninflammatory functional ID, inflammatory functional ID, and hypotransferrinaemia.

*Abbreviations:* ID, iron deficiency; TSAT, transferrin saturation; TIBC, transferrin iron-binding capacity.

*TSAT-Ferritin index:* normal iron status defined as TSAT≥20%, absolute ID as TSAT<20% and ferritin<40 ng/ml, and functional ID as TSAT<20% and ferritin≥40 ng/ml.

*TSAT-TIBC index:* normal iron status defined as TIBC≥50 µmol/L and TSAT≥20%, ID as TIBC≥50 µmol/L and TSAT<20%, inflammatory syndrome as TIBC<50 µmol/L and TSAT<20%, and hypotransferrinaemia as TIBC<50 µmol/L and TSAT≥20%.

**Table 6:** Patient characteristics and measures according to iron status profile.

	Normal	Absolute ID	Noninflammatory functional ID	Inflammatory functional ID	Hypotransferrinaemia	p-value
**No**	538	62	188	21	202	
**Women (%)**	26.8	64.5*	28.7	23.8	24.8	<0.0001
**Age (years)**	60.9±14.3	54.5±17.0*	62.9±12.5	60.9±15.0	57.3±16.2*	<0.0001
for men	60.9±14.4	59.0±15.3	63.0±12.2	62.8±15.1	58.1±16.4*	0.06
for women	60.9±13.8	52.0±17.5*	62.6±13.3	54.9±14.2	54.8±15.4*	0.0005
**Subsaharian African origin(%)**	7.4	6.7	11.0	0.0	10.7	0.5
**Diabetic nephropathy (%)**	11.7	14.5	16.5	19.1	10.9	0.4
**Body mass index (kg/m^2^)**	26.5±4.5	25.6±5.5	27.7±5.8*	27.1±6.0	25.5±4.7*	0.0002
**UPCR (mg/mmol)**	27.3 [12.9–86.4]	30.5 [13.1–87.7]	32.3 [12.5–123.5]	166.9 [26.4–230.0]*	52.8 [16.5–171.2]*	<0.0001
**mGFR mL/min per 1.73 m^2^**	42.3±19.4	41.2±19.4	37.9±17.1*	34.3±19.6*	35.2±18.1*	<0.0001
**C-reactive protein>8 mg/L**	7.3	16.7*	20.9*	45.0*	13.5*	<0.0001
**Albumin (g/L)**	40.4±4.2	38.0±4.0*	38.6±5.1*	35.0±7.2*	38.0±5.8*	<0.0001
**Hb (g/dL)**	13.0±1.5	11.6±1.7*	12.3±1.6*	12.0±1.4*	12.0±1.6*	<0.0001
**WHO anaemia (%)**	42.9	72.6*	59.6*	71.4*	66.3*	<0.0001
Men	42.4	68.2*	61.2*	75.0*	63.8*	<0.0001
Women	44.4	75.0*	55.6	60.0	74.0*	0.0007
**Serum iron (µmol/L)**	16.8±4.5	8.3±2.6*	9.8±2.1*	7.3±1.4*	14.9±4.3*	<0.0001
**Ferritin (ng/mL)**	141 [76–234]	22 [12–31]*	107 [62–178]*	171 [100–217]	164 [104–253]*	<0.0001
**Mean Corpuscular volume (fl)**	90.6±5.7	86.2±6.0*	88.3±5.8*	88.6±4.5	89.8±6.8	<0.0001
**Mean Corpuscular Hb (pg)**	30.3±2.1	28.3±2.4*	29.4±2.2*	29.5±1.9	30.0±2.5	<0.0001
**Hepcidin (ng/mL) No**	97	12	37	4	38	
	29.3 [18.9–42.3]	4.4 [0.7–11.2]*	22.2 [16.1–41.9]	26.1 [6.5–69.1]	35.0 [21.4–50.4]*	<0.0001
**Erythropoietin (IU/L) No**	131	11	47	7	55	
	8.5 (5.8–12.1)	11.6 (8.2–17.6)*	9.4 (7.2–11.7)	7.9 (5.9–10.4)	8.4 (5.5–10.4)	0.08

Data are expressed as means ± SD, median [interquartile range] or %.

* P-value of the comparison with the normal category<0.05.

*Abbreviations:* UPCR, urinary protein-to-creatinine ratio; mGFR, measured glomerular filtration rate; Hb, haemoglobin; WHO anaemia, defined according to World Health Organization as Hb level <13 g/dL for men (<12 g/dL for women); TSAT, transferrin saturation; TIBC, transferrin iron-binding capacity; ID, iron deficiency.

*Definitions:* Normal iron status (TSAT≥20% and TIBC≥50 µmol/L), absolute ID (ferritin<40 ng/mL, TSAT<20%, TIBC≥50 µmol/L), non-inflammatory functional ID (ferritin≥40, TSAT<20, TIBC≥50,), inflammatory functional ID (ferritin≥40, TSAT<20, TIBC<50), and hypotransferrinaemia (TSAT≥20, TIBC<50).

Hepcidin was severely depressed in absolute ID and increased in hypotransferrinaemia, but did not differ from normal for other disorders. In those measured for EPO, its level was significantly increased only in absolute ID (Table 6).

## Discussion

This study showed that a 3-marker index combining ferritin with TSAT and TIBC better demonstrates the impact of the various iron metabolism disorders on Hb levels than individual iron tests or 2-marker indexes do. This is the first study to examine this index and its relation to Hb in a cohort of CKD patients. This combined index revealed two main pathologic mechanisms, namely, non-inflammatory ID and hypotransferrinaemia, and showed that the roles of inflammatory and absolute ID were minor. Moreover, the relations between these iron profiles and decrease in Hb tended to strengthen when mGFR was below 30 mL/min/1.73 m^2^. Our finding that the magnitude of the Hb decrease associated with isolated hypotransferrinaemia was as high as that for other iron profiles provides new insight into CKD anaemia.

Absolute ID was uncommon, but its prevalence depended highly on the ferritin cut-off used to define ID. Using a ferritin level <100 ng/mL and TSAT<20%, Fishbane et al found about 25% of the men with estimated GFR<60 mL/min/1.73 m^2^ had absolute ID, and more than 60% of women, i.e., more than 5 times more than here [5]. While 100 ng/mL is considered the lower acceptable limit in CKD patients, choosing 40 ng/mL for ferritin more specifically identifies patients with ID due to blood loss [12]. We also observed that this cut-off explained Hb variability best. In patients with inflammation, a range of 50 to 100 ng/mL has been suggested for this cut-off [12], [13]. Given that the combined TSAT-TIBC-ferritin index classifies patients with inflammatory iron status apart from the other categories, a low ferritin cut-off value seems more justified for the definition of absolute ID in the combined index. Like others [5], [14], we found absolute ID to be much more common in women. As expected, this profile was associated with the lowest mean value for hepcidin.

Functional ID without inflammation was one of the most frequent iron disorders. Its prevalence steadily increased with CKD progression and its association with Hb decline also strengthened. Compared with inflammatory ID, this category included patients with normal transferrin, lower C-reactive protein level and higher albuminemia. On the other hand, inflammatory ID was relatively rare in our cohort. The prevalence we observed was similar to the 3% prevalence of inflammatory iron status evaluated by bone marrow parameters in non-dialysis CKD patients [4]. However, from a clinical point of view, its prevalence is high during acute complications. Inflammation affects Hb *via* the iron pathway. Adjustment for CRP controlled for the impact of inflammation *via* the non-iron pathway. An increase of hepcidin with CKD decline and inflammation was expected to be one of the main mechanisms implicated in these functional ID profiles [15]. However, we failed to find any association between hepcidin and these profiles.

The second most frequent pathologic iron status was hypotransferrinaemia with both normal TSAT and ferritin. This category was created to separate the 202 patients (19.9%) with isolated low transferrin from those with all normal iron tests. An original finding of this study is that this category is associated with an Hb decrease as low as in the three other categories, for it validates the abnormality of isolated hypotranferrinaemia. The decreased transferrin level may have misleadingly normalized TSAT, but serum iron and ferritin level were noticeably high in this patient group and reflected normal iron reserves. This iron profile was also seen in hemodialysis patients [16]. Isolated hypotransferrinaemia would be mostly secondary to malnutrition [17] and urinary loss [18] and different from that observed in inflammatory ID. This hypothesis is consistent with the lower BMI and albuminaemia levels in patients with hypotransferrinaemia compared with normal profiles, together with a strong proteinuria increase and only a slight CRP increase compared to the inflammatory ID profile. In addition, a decrease in bone marrow activity may modify the transferrin metabolism [19]. Transferrin decreased as GFR fell in our cohort. This finding corroborates the results of the MDRD study [20]. Protein and energy intake did not fully explain the transferrin trends in that study, although they did explain other nutritional parameters, such as albuminaemia [20]. Nutrition status and bone marrow activity, which both worsen with CKD progression, can influence transferrin levels.

To our knowledge, a high prevalence of isolated hypotransferrinaemia has never before been shown in CKD patients, nor its association with anaemia. Malnutrition might be the underlying cause of anaemia in this group, but hypotransferrinaemia may also play a direct role in anaemia occurrence. A transferrin deficiency can induce anaemia *per se* as seen in human atransferrinaemia and in animal models [21], [22]. Atransferrinaemia is a rare autosomal recessive disease that causes hypochromic anaemia [23]. These patients have no iron staining in the blood marrow but nonetheless have iron overload in the liver and spleen. Iron normally internalized *via* the transferrin pathway cannot be delivered to erythrocyte precursors. Plasma or purified apotransferrin infusions normalize their Hb levels [24] and increase their hepcidin concentrations [25], [26]. Patients with hypotransferrinaemia in our cohort share some features of human atransferrinaemia and of the hypotransferrinaemia animal models, in particular, normal serum iron and normal to high iron stores. However, in contrast to animal models, the hepcidin level was not below normal in this patient group, but rather slightly above normal. Given the cross-sectional design of our study and the short lifetime of hepcidin, it is possible that we missed a transient phase of its decrease.

The major strengths of our study include its large sample size of well-phenotyped patients with a wide range of renal function, a large number of laboratory measurements, and its use of measured GFR. The study power was sufficient to conduct subgroup analyses according to mGFR and to show significant associations with Hb changes for all iron status profiles, except the small group of 21 patients with inflammatory ID. The higher number of men than women in this cohort reflects the well-established higher risk for CKD in men, but the lack of interaction with gender in the associations between iron status and Hb allows us to apply results to both genders.

This study also has limitations, however, related to its cross-sectional design that prevents causal inferences. For example, whether hypotransferrinaemia truly caused hepicidin to increase or was preceded by a decrease in hepcidin cannot be answered. The second limitation is linked to the basic evaluation of iron metabolism. Iron stores can be evaluated more accurately in bone marrow; erythrocyte precursor iron uptake is influenced by transferrin receptors and can be assessed more exactly by the percentage of hypochromic reticulocytes. Those are however less frequently used, and our purpose was to show how we may improve the use of routine iron tests in CKD anaemia.

Altogether, the TSAT-TIBC-ferritin index developed in this study clarifies the different iron metabolism disorders at work in CKD anaemia. It was shown to be associated with decreased Hb levels more strongly than either each iron marker taken separately or previous combined iron indexes. This index showed non-inflammatory functional ID and hypotransferrinaemia to be the major mechanisms of iron disorders in CKD anaemia. These findings should encourage clinical trials to study iron therapy and ESA responsiveness according to patient iron profile. We may hypothesize, for example, that higher iron stores and intravenous iron delivery could be necessary for patients with hypotransferrinaemia to facilitate erythrocyte precursor iron uptake. This may further influence the management of iron therapy in CKD anaemia. Responsiveness to ESA might also differ according to iron profiles. Morbidity/mortality rates are higher for patients with high ESA doses. In particular, the safety and utility of increasing these ESA doses might vary by iron profile.

Information about access to the NephroTest data appears on the French website describing all on-going cohorts in France: https://epidemiologie-france.aviesan.fr/catalog/search.jsp

## Supporting Information

Figure S1 **Distribution of Hb, transferrin saturation (TSAT), ferritin and transferrin iron binding capacity (TIBC).**
(TIF)
