# Correction: A 3-Marker Index Improves the Identification of Iron Disorders in CKD Anaemia

**DOI:** 10.1371/journal.pone.0100606

**Published:** 2014-06-12

**Authors:** The PLOS ONE Staff

There is an error in Table 6. The last two columns of the first row were shifted into the second row during the typesetting process. Please see the corrected Table 6 below.

**Table 6:** Patient characteristics and measures according to iron status profile.

	Normal	Absolute ID	Noninflammatory functional ID	Inflammatory functional ID	Hypotransferrinaemia	p-value
**No**	538	62	188	21	202	
**Women (%)**	26.8	64.5*	28.7	23.8	24.8	<0.0001
**Age (years)**	60.9±14.3	54.5±17.0*	62.9±12.5	60.9±15.0	57.3±16.2*	<0.0001
for men	60.9±14.4	59.0±15.3	63.0±12.2	62.8±15.1	58.1±16.4*	0.06
for women	60.9±13.8	52.0±17.5*	62.6±13.3	54.9±14.2	54.8±15.4*	0.0005
**Subsaharian African origin(%)**	7.4	6.7	11.0	0.0	10.7	0.5
**Diabetic nephropathy (%)**	11.7	14.5	16.5	19.1	10.9	0.4
**Body mass index (kg/m^2^)**	26.5±4.5	25.6±5.5	27.7±5.8*	27.1±6.0	25.5±4.7*	0.0002
**UPCR (mg/mmol)**	27.3 [12.9-86.4]	30.5 [13.1-87.7]	32.3 [12.5-123.5]	166.9 [26.4-230.0]*	52.8 [16.5-171.2]*	<0.0001
**mGFR mL/min per 1.73m^2^**	42.3±19.4	41.2±19.4	37.9±17.1*	34.3±19.6*	35.2±18.1*	<0.0001
**C-reactive protein> 8 mg/L**	7.3	16.7*	20.9*	45.0*	13.5*	<0.0001
**Albumin (g/L)**	40.4±4.2	38.0±4.0*	38.6±5.1*	35.0±7.2*	38.0±5.8*	<0.0001
**Hb (g/dL)**	13.0±1.5	11.6±1.7*	12.3±1.6*	12.0±1.4*	12.0±1.6*	<0.0001
**WHO anaemia (%)**	42.9	72.6*	59.6*	71.4*	66.3*	<0.0001
Men	42.4	68.2*	61.2*	75.0*	63.8*	<0.0001
Women	44.4	75.0*	55.6	60.0	74.0*	0.0007
**Serum iron (ìmol/L)**	16.8±4.5	8.3±2.6*	9.8±2.1*	7.3±1.4*	14.9±4.3*	<0.0001
**Ferritin (ng/mL)**	141 [76-234]	22 [12-31]*	107 [62-178]*	171 [100-217]	164 [104-253]*	<0.0001
**Mean Corpuscular volume (fl)**	90.6±5.7	86.2±6.0*	88.3±5.8*	88.6±4.5	89.8±6.8	<0.0001
**Mean Corpuscular Hb (pg)**	30.3±2.1	28.3±2.4*	29.4±2.2*	29.5±1.9	30.0±2.5	<0.0001
**Hepcidin (ng/mL) No**	97	12	37	4	38	
	29.3 [18.9-42.3]	4.4 [0.7-11.2]*	22.2 [16.1-41.9]	26.1 [6.5-69.1]	35.0 [21.4-50.4]*	<0.0001
**Erythropoietin (IU/L ) No**	131	11	47	7	55	
	8.5 (5.8-12.1)	11.6 (8.2-17.6)*	9.4 (7.2-11.7)	7.9 (5.9-10.4)	8.4 (5.5-10.4)	0.08

Data are expressed as means ± SD, median [interquartile range] or %

* P-value of the comparison with the normal category<0.05.

*Abbreviations:* UPCR, urinary protein-to-creatinine ratio; mGFR, measured glomerular filtration rate; Hb, haemoglobin; WHO anaemia, defined according to World Health Organization as Hb level <13 g/dL for men (<12 g/dL for women); TSAT, transferrin saturation; TIBC, transferrin iron-binding capacity; ID, iron deficiency.

*Definitions:* Normal iron status (TSAT ≥ 20% and TIBC ≥ 50 µmol/L), absolute ID (ferritin <40 ng/mL, TSAT<20%, TIBC≥50 µmol/L), non-inflammatory functional ID (ferritin≥40, TSAT<20, TIBC≥50,), inflammatory functional ID (ferritin≥40, TSAT<20, TIBC<50), and hypotransferrinaemia (TSAT≥20, TIBC<50)
